# Online Peer-to-Peer Mentoring Support for Youth with Hemophilia: Qualitative Needs Assessment

**DOI:** 10.2196/10958

**Published:** 2018-07-10

**Authors:** Vicky R Breakey, Vanessa Bouskill, Cynthia Nguyen, Stephanie Luca, Jennifer N Stinson, Sara Ahola Kohut

**Affiliations:** ^1^ Division of Pediatric Hematology/Oncology Department of Pediatrics McMaster Children’s Hospital Hamilton, ON Canada; ^2^ Faculty of Health Sciences McMaster University Hamilton, ON Canada; ^3^ Division of Hematology/Oncology Department of Pediatrics The Hospital for Sick Children Toronto, ON Canada; ^4^ Lawrence S Bloomberg Faculty of Nursing University of Toronto Toronto, ON Canada; ^5^ Child Health Evaluative Sciences The Hospital for Sick Children Toronto, ON Canada; ^6^ Department of Anesthesia and Pain Medicine The Hospital for Sick Children Toronto, ON Canada; ^7^ Department of Psychiatry The Hospital for Sick Children Toronto, ON Canada

**Keywords:** hemophilia, adolescents, transition, self-management, education, internet, mentoring

## Abstract

**Background:**

To support adolescents through transition from pediatrics to adult care, health care providers and families help teens gain knowledge and develop self-management skills. Peer mentoring can provide meaningful support and has been associated with improved health outcomes in patients with other chronic conditions. Peer mentoring is an appealing way to provide support, but it is imperative to consider the unique needs of adolescents to ensure its success.

**Objective:**

The objective of our study was to identify the peer mentoring wants and needs of youth with hemophilia in order to guide the development of a new program.

**Methods:**

In this qualitative study, we interviewed a convenience sample of youth with hemophilia from 2 Canadian hemophilia treatment centers. Two iterative cycles of audiorecorded, semistructured individual interviews were conducted. Descriptive statistics and content analyses were used to organize data into categories that reflected emerging themes.

**Results:**

In total, we recruited 23 participants aged 12-20 years, with a mean age of 14.91 (2.57) years. When asked about program design, participants weighed the importance of flexibility in delivery (eg, Web-based, in person, text messaging [short message service]), content (eg, structured vs unstructured), frequency of sessions, and length of the program. Participants identified some potential challenges such as scheduling issues, comfort level for disease discussion, and discordant mentor-mentee personality types. The program was viewed as a positive medium for connecting peers with hemophilia.

**Conclusions:**

Adolescents with hemophilia expressed interest in a peer mentoring program and provided valuable insight that will be applied in the development of a peer mentoring program for youth with hemophilia.

## Introduction

The challenges of transition of care for young people with hemophilia have been documented and well reviewed [1-3]. As the responsibilities of disease management shift from caregivers to patients themselves, adolescents are expected to gain knowledge and self-management skills to become independent. Despite well-developed resources for North American hemophilia care providers [4-6], adolescents and young adults report gaps in the understanding and skills [7-9].

Recently, a pilot study suggested that a Web-based learning program is an effective tool for providing education and self-management skills to youth with hemophilia [10]. Following completion of the program, adolescents made significant gains in knowledge, self-efficacy, and readiness for transition of care from caregivers to self. One aspect of the program that was highly rated by adolescents was the support gained from a trained “health coach,” who made weekly telephone calls to discuss their progress and answer their questions. Although this element was popular with adolescents, providing this one-on-one support is challenging as it requires significant ongoing resources.

Peer mentoring support in health care is an explicit form of social support established to provide individuals with emotional (eg, expressions of caring, empathy, and reassurance), appraisal (eg, affirmation of one’s feelings and behaviors, encouraging persistence for conflict resolution, and reassurance that frustrations can be handled), and informational (eg, providing advice, suggestions, and facts relevant to issues with which the peer is dealing) support from other people living with similar conditions [11]. Peer mentoring can provide meaningful social support and has also been associated with improved health outcomes [12-15]. In qualitative interviews, some adolescents with hemophilia expressed feelings of isolation and voiced interest in obtaining peer support; older adolescents and young adults felt that peer support was valuable and expressed a desire to mentor younger youth [7].

A promising Skype-based peer mentoring support intervention (iPeer2Peer Program; iP2P) has been piloted in youth with arthritis and chronic pain. The program sex matched young adults (mentors) who had successfully transitioned to adult care with adolescents aged 12-18 years (mentees), and they completed 10 Skype calls over an 8-week period. When compared with a waitlist control group, youth demonstrated improvements in self-management and pain-coping efficacy immediately after completing iP2P [16,17]. As arthritis and chronic pain affect more females than males, only a few males participated in the study. Given that hemophilia is X-linked, it is important to explore whether an adaptation of the iP2P program would enhance the self-management and transitional care in this population.

In order to explore the need for and acceptability of an iP2P peer mentorship program for youth with hemophilia, we conducted a needs assessment with qualitative interviews. The aims of the needs assessment were to (1) document the perceived self-management needs of youth with hemophilia, (2) determine if Web-based peer mentoring is an acceptable means of learning self-management strategies, and (3) gather information on youth preferences and ideas for an optimal Web-based mentoring program, if developed.

## Methods

### Participant Recruitment Strategy

A qualitative descriptive study using semistructured individual interviews was conducted in 2015-2016 with adolescents and young adults living with moderate or severe hemophilia from 2 tertiary hemophilia treatment centers (HTCs) in Ontario (The Hospital for Sick Children, Toronto, and McMaster University Medical Centre, Hamilton). A purposive sampling method was used based on age to maximize the variability of the sample. Eligibility inclusion criteria included adolescents and young adults who were (1) between 12 and 25 years old, (2) diagnosed with moderate or severe hemophilia, (3) able to speak and comprehend English, and (4) willing and able to complete an in-person or telephone interview. Exclusion criteria included (1) a significant cognitive delay and (2) major comorbid illnesses (medical or psychiatric). The age range was chosen to foster “near peer” relationships between potential mentors and mentees. The eligibility was determined upon review of the local patient database by the clinical team. Research ethics boards at both hospitals approved the study.

### Interview Protocol

Once informed consent was obtained, participants completed demographic questionnaires. Interviews followed a semistructured format using an interview guide developed by the study team based on clinical experience and current research literature. Participants were asked about their experiences living with hemophilia, their interests in meeting other youth with hemophilia, and being involved in a Skype-based peer mentoring program, as well as the features they would want in a new program (eg, frequency, length, and content).

### Statistical and Qualitative Analyses

Descriptive statistics were used to summarize sample characteristics. Audiotaped interviews were transcribed verbatim and verified against the audio recordings and notes taken during the interviews. Transcribed data were imported into NVivo 10 [18]. Under the supervision of a coinvestigator (JNS), the analyses were conducted independently by two members of the research team (SL, CN) and two research students using simple content analyses, as outlined by Sandelowski [19]. The study team reviewed a subset of the transcripts, and preliminary themes were used to develop and revise the coding scheme through discussion. To ensure the coding scheme was grounded in the data, raw data were revisited throughout the analytic process [20]. Interrater reliability analyses were conducted to establish agreement between coders.

## Results

### Study Participants

Across the 2 tertiary HTCs, 56 patients were approached. Of those approached, 28 patients declined due to lack of interest in the study and 28 patients were consented and enrolled. However, 5 participants were lost to follow-up during the interview phase (ie, could not be contacted via telephone or email); thus, 23 participants completed the study. Demographic characteristics for the adolescent and young adult sample are summarized in Table 1.

All 23 participants had a computer at home with internet access. Of all, 56% (13/23) participants spent over 7 hours on the internet and 47% (11/13) spent over 7 hours on the computer each week. All participants reported being “comfortable” or “very comfortable” using the computer and the internet. Computer usage data were missing for 2 participants.

### Thematic Analysis for Disease Impact

Thematic analysis revealed five major disease impact themes: physical, emotional, social, school and work, and the future. Major themes and key quotes from participants are summarized in Table 2.

### Thematic Analysis for Program Development

Participants were asked for their opinions about a mentorship program for adolescents with hemophilia. Thematic analysis revealed six major themes for program development: content, delivery, frequency, length, potential challenges, and anticipated benefits. We have summarized major themes and participant comments.

#### Content

Participants described wanting mentorship on the challenges of living with hemophilia, including different treatments, self-management experiences, and sports participation. Opinions differed on whether conversation should be guided by pre-set questions or be unstructured. For example:

I think it should really be whatever you want to talk about because if it is structured, then it’s more like a robot interaction...but if you personalize it you make it more free-flowing then...it accomplishes more.

Age 14

Most participants agreed that the mentorship relationship should begin by discussing general topics and then progress with more personal topics, as mentees grew more comfortable with the mentor where trust and rapport had then been developed.

**Table 1:** Demographic characteristics of the adolescent and young adult sample.

Characteristic		Adolescents and young adults (n=23)
**Sex, n (%)**		
	Male	23 (100)
	Female	0 (0)
Age, mean (SD)		14.91 (2.57)
**Current level of education^a^, n (%)**		
	Grade 7	6 (26)
	Grade 8	3 (13)
	Grade 9	2 (9)
	Grade 10	1 (4)
	Grade 11	4 (18)
	Grade 12	3 (13)
	University	1 (4)
	College	1 (4)
**Ethnicity, n (%)**		
	White	15 (64)
	Black	2 (9)
	Japanese	1 (4)
	Latin American	1 (4)
	South Asian	1 (4)
	South East Asian	1 (4)
	Other	2 (9)
	Do not want to answer	0 (0)

^a^Data related to current level of education is missing for 2 participants.

**Table 2:** Impact of hemophilia on teens and young adults.

Disease Impact	Major Findings	Exemplar Quotes
Physical	Impact of pain associated with bleeds and with regular venipuncture Some perceived minimal physical impact largely because hemophilia is easily treated by prophylactic treatment Some learned about physical risks through individual experiences and seeking information from their health care team, family, or friends with hemophilia	*Well its actually been quite a challenge, always getting needles probably like every Tuesday and Thursday...it’s been kinda difficult. [Age 12]*
		*I sprained my ankle when I was in grade 4...[that joint] started to bleed a lot...there was more risk involved, like cartilage problems [and] like chronic pain...which is not something that would easily happen to someone without hemophilia because it clots. [Age 14]*
		*Well, luckily, I did have an older brother and I did learn a bit from his mistakes, but I mean obviously, you want to try things. [Age 17]*
Emotional	Main cause of emotional upset was not being able to play sports or having to miss out on activities or extracurriculars	*It’s impacted my life a lot, sometimes it’s hard to deal with. I can’t play any contact sports, and sometimes seeing your friends playing sports...it’s definitely an experience, but you do learn to deal with it. [Age 17]*
		*Everyone kept telling me...you’re very fragile, in fact, when I was a kid, I felt more like a glass vase...because I was afraid that I would break out to a bleed. [Age 18]*
		*I can’t really do much...like...a lot sports, like hockey or football, because if I get hurt really badly I would end up in the hospital. [Age 12]*
Social	Inability to participate in certain activities limited social interaction with friends Feelings of isolation because participants did not feel comfortable explaining their condition to peers Having hemophilia gave some participants an opportunity to create strong bonds with family members and peers who have the same condition	*Probably the only one big challenge that I have faced is like doing stuff with friends. Like sometimes friends are doing stuff that I wouldn’t participate in and it would feel a little bit sad...I just kind of accepted it and my friends have been really understanding about it. [Age 14]*
		*You felt sometimes you didn’t want to tell people if you had it...you didn’t want people to treat you differently...I kind of kept to myself when I was really young. [Age 20]*
		*I have a younger brother he also has hemophilia. We connected [and] it was great...With my parents...I got to see how caring they were. They were out almost all day out at the hospital rooms or emergencies all the time. [Age 20]*
School and Work	Majority felt having hemophilia did not impact their school attendance, as teachers were accommodating and supportive, but some decreased their participation in physical education due to risks Of those currently employed, most did not disclose their condition as they felt it did not interfere with their position and there was no high risk of injury at work	*Well in school, in gym, sometimes I have to skip out on some of the activities, because they’re a little too...rough for me to play. [Age 14]*
		*Yea, my boss knows I have hemophilia, she understands what’s happening, she’s you know um, considerate about it, she understands I won’t be able to work for a while. [Age 17]*
Future	Most did not envision hemophilia having an impact on their career path, aside from avoiding physically demanding careers Expressed a need to choose postsecondary institutions where easy access to medical services was available No major concerns about hemophilia affecting future romantic relationships or having children	*I’m pretty sure it won’t affect my future career, but it could limit my career, like since I can’t do much, most of the hard work. [Age 17]*
		*Going to the future...if I do have kids, you know the females would be carriers and the males would be unaffected that’s fairly okay. I don’t mind passing on the gene. I could deal with that. [Age 20]*

#### Delivery

Participants varied in their preferences of delivery method of mentorship (eg, in person, Web-based, or telephone or texting). In-person mentorship sessions were felt to be most convenient if they were held on the same day as a regular clinic appointment. Web-based mentorship (eg, Facetime or Skype) was viewed as convenient for several reasons, including not having to rely on anyone for transportation, for individuals who live far away from the hospital, and the flexibility to schedule sessions at convenient times and minimize scheduling conflicts. Participants also reflected that they might also be more willing to open up to someone online, rather than in person. Some participants mentioned texting as a good option for those who did not like face-to-face interactions; however, others indicated that it would be difficult to connect with their mentors solely through texting. There were also varying opinions about individual versus group-based mentorship. Some participants stated that they would feel intimidated and uncomfortable sharing personal information in a group, even on an online group forum. However, others saw value in hearing from a group to obtain a broader perspective and stated that it would increase the likelihood of connecting with peers in the group. One teen stated the following:

I think both are good. Both have pros and cons. Um if it is a group discussion, I don’t think they would be sharing that much information about private stuff though but uh depends on the person. But I think most people will probably like one-on-one.

Age 20

#### Frequency

Participants’ preferences for the frequency of mentoring sessions ranged from twice a week to bimonthly. Some participants preferred not to have scheduled sessions, but rather only have sessions as needed. Some felt they would find comfort in knowing that they could have a mentor to reach out to when needed. Some participants indicated that the frequency of sessions would depend on whether sessions were held in person or were online. A teen said the following:

I would say once a week or, if it was at my home, probably twice a week. Just because [the hospital] is a commute every time to get there.

Age 13

#### Length

Participants also indicated a range for length of sessions, from 5 minutes to 2 hours. Some participants indicated that they preferred not to have a time constraint placed on them, but rather have the session be as short or as long as they needed. A participant said the following:

I think it all just depends on the person themselves...whether they have a lot of questions or not, I guess the ideal time would be around 20 minutes...longer if they have more questions.

Age 17

#### Potential Challenges

When asked, only a few participants could list the potential challenges to the feasibility and uptake of a peer mentoring program (Table 3).

One potential challenge was shyness or lack of openness among mentees. Participants were also worried there might be a personality clash or difficulty establishing a connection with the mentor. Some participants were concerned that mentees would not know how to ask the right questions and that finding experienced individuals to fill the mentor role may be a challenge.

Another challenge related to a group-based mentorship program was a concern about hearing about other teens’ struggles and problems. Some thought that this would pose an unnecessary burden on them. Timing of the program was also seen as a challenge, with participants generally preferring to have a mentor at an age when they are starting to take on more disease self-management responsibilities or struggling to manage symptoms or treatment.

#### Anticipated Benefits

When asked about the possible benefits of receiving mentorship, one of the biggest perceived benefits was feeling like they are not alone in their disease. They indicated that participating in a mentoring program would help them feel being part of the hemophilia community and gain a sense of hope seeing someone with hemophilia succeeding in adulthood. Older participants expressed an interest in being a mentor as they felt confident in their ability to manage their condition independently.

**Table 3:** Challenges of a peer mentoring program for adolescents with hemophilia.

Challenges		Exemplar quotes
**Connection**		
	Issues establishing rapport between mentors and mentees (eg, shyness, comfort level, personality)	*[What if a] mentee just doesn’t feel like a connection to the person that they’re talking to, they don’t open up to them, or if the child doesn’t know what questions to ask the peer mentor. [Age 17]*
**Timing of program delivery**		
	Prefer to access program when starting to take on more disease self-management responsibilities or struggling to manage symptoms or treatment	*It helps little kids to prepare for like, how to do their needles, or to prepare for how it’s going to affect their life later on in the future. [Age 14]*
**Scheduling**		
	Difficult to find a time when all parties are available; especially difficult if sessions occur in person or in a group setting	*I guess finding the time to all meet up and all that. If it’s just like a big group. [Age 13]*
**Time commitment**		
	Full schedule of extracurricular activities make it difficult to find time to fit in another activity	*People have to do a lot of things...time is on the shortage most of the time, most people don’t have enough time to like get to other people. [Age 17]*
**Slow enrollment**		
	Concerns about finding enough adolescents to participate due to lack of interest	*But I guess just trying to get the kids interested...I think that would be hardest part, having kids actually want to...participate without them having it enforced upon them. [Age 20]*

**Table 4:** Benefits of a peer mentoring program for adolescents with hemophilia.

Benefits		Exemplar quotes
**Sense of community**		
	Feeling like they are not alone in their disease	*I think [the mentorship sessions] would make kids more open about hemophilia...talk about it, talk to their friends about it. Making them feel like they’re not alone you could say like they’re not the only ones. Inclusion. [Age 20]* *People really having a sense of community about their hemophilia because alone we are just hemophiliacs but together we are a social group, we are a community that’s helping one another. [Age 14]*
**Sense of hope**		
	Mentor success in adulthood	*They’ll also feel like there’s someone in the same boat as me...cause you almost feel isolated, when no one else has it...So if they have someone to talk to...[they look] forward to the future and it’s not just going to be a boring future. [Age 17]*
**Opportunity for discussion**		
	Speak about hemophilia from a nonmedical perspective; focus on aspects of daily life	*Well, I would, like, learn a lot through someone that is more skilled, who would understand more than I do. [Age 12]*
**Ease of dialogue**		
	Extension of attending hemophilia summer camp; easier to speak to someone of a similar age and diagnosis	*Definitely just someone to talk to about hemophilia would awesome because you can just talk to them about things in your life that are affected by hemophilia...you can relate to them because they’ve gone through what you’ve been through. [Age 14]*

Many participants made reference to attending a hemophilia summer camp and saw the mentoring program as a welcome extension of the camp. Most participants also indicated that it would be easier to speak to someone with a similar age and diagnosis. Some participants indicated that they would find the mentoring sessions helpful because they would have the opportunity to speak about how hemophilia has affected their lives and to ask for advice about hemophilia management and about impact of hemophilia on other aspects of typical teenage life. A summary of perceived benefits of a mentoring program is shown in Table 4.

## Discussion

Not surprisingly, our subjects reported that their hemophilia has had significant effects on the physical, emotional, and social aspects of their lives. The challenges of activity or sports restrictions permeated this dialogue, suggesting this to be the most significant stressor facing our young people. Other voiced challenges involved feelings of isolation and discomfort among participants related to disclosure of their condition. These results were similar to those of previously published studies [9,21], suggesting that these issues are universal and reinforcing the areas in which young people with hemophilia require support.

In addition to common challenges, our participants shared a strong interest in gaining support through mentorship from more experienced individuals who were living with the condition. Many discussed their experiences at hemophilia camp as a time when they met others and received support. Thomas and Gaslin described the importance of such camps as means to improve self-esteem in young people with hemophilia [22]. In addition, a recent systematic review suggested that participation in the camps had therapeutic effects on the aspects of health-related quality of life [23]. Discussions with our subjects about the benefits provided by hemophilia camp support an expansion of available mentorship to include youth that are not able to attend summer camp, as well as to extend the supports gained through camping programs into the school year through an alternative program.

In preparing to develop a new mentorship initiative, we recognized the value of asking for input from potential mentees and mentors prior to building the program. We used the interview platform to determine participants’ views on peer support and the acceptability of Web-based mentorship. Further areas of the program can be explored in future studies (eg, age preference of mentors, life experiences). While we recognized that published data have shown this to be a feasible and satisfying approach for patients with other chronic conditions [17], we wanted to ensure that this approach could be applied to our patients with hemophilia and tailored it to their specific needs as necessary. No formal comparison has been made between the needs of patients with hemophilia and those with other chronic conditions where a peer mentorship program has been piloted [16,17]. We anticipate that the needs of patients with hemophilia would be different due to the condition being present at birth, which may impact their perspectives and management of their conditions. In addition, treatment options vary across populations.

The limitations of this study include a relatively small study population in 2 proximal centers. The sample size was small, but in keeping with an appropriate size for this qualitative methodology [24]. Content themes were saturated, suggesting that central issues were identified. Although the results may not be completely generalizable, significant insight has been provided into the thoughts and experiences of young people with hemophilia. Although a purposive sampling method was used, there is a potential for selection bias in favor of youth who are either motivated to participate or interested in a peer support program. The invitation to participate was extended to all eligible participants, and the decision to partake was voluntary.

In summary, it is essential to consider end-user needs and preferences prior to developing support programs to ensure that we meet the needs of our patients. While our participants generally supported Web-based peer mentorship, our data suggest that there is no one-size-fits-all approach that will meet the needs of adolescents and young adults with hemophilia. Through the data obtained, we were able to catalogue the perceived needs of this patient population and document its preferences and opinions on how best to develop a Web-based mentoring program. Moving forward, we will aim to build a program that is flexible and teen driven with a supportive backbone of education and social supports.

## Abbreviations

HTC: hemophilia treatment center
iP2P: iPeer2Peer Program
